# Vimar Is a Novel Regulator of Mitochondrial Fission through Miro

**DOI:** 10.1371/journal.pgen.1006359

**Published:** 2016-10-07

**Authors:** Lianggong Ding, Ye Lei, Yanping Han, Yuhong Li, Xunming Ji, Lei Liu

**Affiliations:** 1 State Key Laboratory of Membrane Biology, School of Life Sciences, Peking University, Beijing, China; 2 Aging and Disease lab of Xuanwu Hospital and Beijing Institute for Brain Disorders, Capital Medical University, Youanmen, Beijing, China; Max Planck Institute for Biology of Ageing, GERMANY

## Abstract

As fundamental processes in mitochondrial dynamics, mitochondrial fusion, fission and transport are regulated by several core components, including Miro. As an atypical Rho-like small GTPase with high molecular mass, the exchange of GDP/GTP in Miro may require assistance from a guanine nucleotide exchange factor (GEF). However, the GEF for Miro has not been identified. While studying mitochondrial morphology in *Drosophila*, we incidentally observed that the loss of *vimar*, a gene encoding an atypical GEF, enhanced mitochondrial fission under normal physiological conditions. Because Vimar could co-immunoprecipitate with Miro *in vitro*, we speculated that Vimar might be the GEF of Miro. In support of this hypothesis, a loss-of-function (LOF) *vimar* mutant rescued mitochondrial enlargement induced by a gain-of-function (GOF) *Miro* transgene; whereas a GOF *vimar* transgene enhanced *Miro* function. In addition, *vimar* lost its effect under the expression of a constitutively GTP-bound or GDP-bound Miro mutant background. These results indicate a genetic dependence of vimar on Miro. Moreover, we found that mitochondrial fission played a functional role in high-calcium induced necrosis, and a LOF *vimar* mutant rescued the mitochondrial fission defect and cell death. This result can also be explained by vimar's function through Miro, because Miro’s effect on mitochondrial morphology is altered upon binding with calcium. In addition, a *PINK1* mutant, which induced mitochondrial enlargement and had been considered as a *Drosophila* model of Parkinson’s disease (PD), caused fly muscle defects, and the loss of *vimar* could rescue these defects. Furthermore, we found that the mammalian homolog of Vimar, RAP1GDS1, played a similar role in regulating mitochondrial morphology, suggesting a functional conservation of this GEF member. The Miro/Vimar complex may be a promising drug target for diseases in which mitochondrial fission and fusion are dysfunctional.

## Author Summary

Mitochondrial dynamics including fusion, fission and transport are essential for energy supply in eukaryotic cells; and defects in mitochondrial dynamics often result in premature aging and diseases such as Parkinson's disease (PD). In mitochondrial transport machinery, the Miro/Milton complex loads mitochondria onto microtubule through kinesin motor proteins; and regulates mitochondrial fusion and fission through unknown mechanisms. As a small GTPase, the exchange of GTP/GDP in Miro requires a specific guanine nucleotide exchange factor (GEF). However, the GEF for Miro has not been identified. In this study, we identified Vimar as a new regulator of mitochondrial dynamics in *Drosophila*. We found that loss of *vimar* promoted mitochondrial shortening; and this function was mediated through Miro. As a GEF, Vimar partially localized on mitochondria and could physically interact with Miro. In the pathophysiological conditions, including a *Pink1* mutant to model PD and a calcium-overload induced stress to model neuronal necrosis in *Drosophila*, loss of *vimar* suppressed both aberrant mitochondrial fusion and fragmentation in PD and necrosis, respectively. As the mammalian homolog of Vimar, RAP1GDS1 function was similar to Vimar. Therefore, Vimar/ RAP1GDS1 may be a great drug target to deal with diseases caused by defective mitochondrial dynamics.

## Introduction

Mitochondrial fission, fusion and transport play important roles for the function of this organelle [1, 2]. The balance between fusion and fission controls mitochondrial morphology, which is mediated by series of large dynamin-related GTPases [3]. Among these GTPases, mitofusin1/mitofusin2 (MFN1/MFN2) and optic atrophy protein1 (OPA1) are the core components that are responsible for mitochondrial fusion [4–7], whereas dynamin-related protein 1 (Drp1) is the core component that is responsible for mitochondrial fission [8, 9]. In addition to these GTPases in dynamin-related family, mitochondrial Rho (Miro), an atypical member of the Rho small GTPase family, has a well-known function of transporting the mitochondria along microtubules [10, 11]. Miro also regulates mitochondrial morphology via inhibition of fission under physiological Ca^2+^ conditions, although the mechanism is not that clear [12–16]. Large GTPases such as dynamin-like GTPase family members hydrolyze GTP and exchange GTP and GDP without the assistance from other regulators [17, 18]. However, members of the small GTPase family often require other proteins to help release their tightly bound GDP or enhance their low GTPase activities. These proteins are referred to as guanine nucleotide exchange factors (GEFs) and GTPase activating proteins (GAPs), respectively [19]. To date, most small GTPases require unique GEFs or GAPs [19].

An understanding of the regulation of mitochondrial dynamics may help us to address many human diseases. For instance, mutations in OPA1 or MFN2 result in dominant optic atrophy or Charcot-Marie-Tooth neuropathy type 2A [20, 21]. Abnormal mitochondrial fission also promotes aging and cell death [22, 23]. In necroptosis, the formation of the necrosome promotes mitochondrial fission through dephosphorylation of Drp1 [24]. In neuronal excitotoxicity, calcium ions are overloaded, resulting in reduced levels of the MFN2 protein, which enhances mitochondrial fission and leads to neuronal necrosis [25, 26]. In addition, other components such as Miro may participate in this process [26]. Miro has two EF hand motifs that bind calcium; thus, Miro can couple calcium increase with reduced mitochondrial motility to meet the locally increased energy demands [16, 27]. Interestingly, Miro also promotes fission in the presence of excess calcium, which is distinct from its inhibitory role in fission under normal calcium concentrations [16]. It is unclear whether Miro plays a functional role in neuronal necrosis [26].

The mitochondrial morphology represents a transient balance between mitochondrial fusion and fission [28]. Using a systematic genetic screen in yeast covering approximately 88% of genes, 117 genes that regulate mitochondrial morphology were identified [29]. Similarly, a screen of 719 genes that are predicted to encode mitochondrial proteins in worms demonstrated that more than 80% of these genes regulate mitochondrial morphology [30]. Although many genes may regulate mitochondrial morphology, their relationships to the core mitochondrial fusion and fission components are unclear.

In studying mitochondrial morphology, we accidently discovered that the loss of *vimar* (visceral mesodermal armadillo-repeats), which encodes an atypical GEF [31–33], promoted mitochondrial fission in *Drosophila* flight muscle cells. Furthermore, we found that vimar was capable of interacting with Miro *in vitro*. Genetically, vimar required normal GDP- or GTP-bound activity of Miro to affect mitochondrial morphology, suggesting vimar is likely the Miro GEF. In addition, we found that the Miro/vimar complex suppressed mitochondrial fission during necrosis and mitochondrial fusion in *PINK1* mutant model of Parkinson’s disease (PD), making vimar a potential drug target.

## Results

### Vimar is a novel regulator of mitochondrial morphology in *Drosophila* under normal conditions

**Fig 1:**
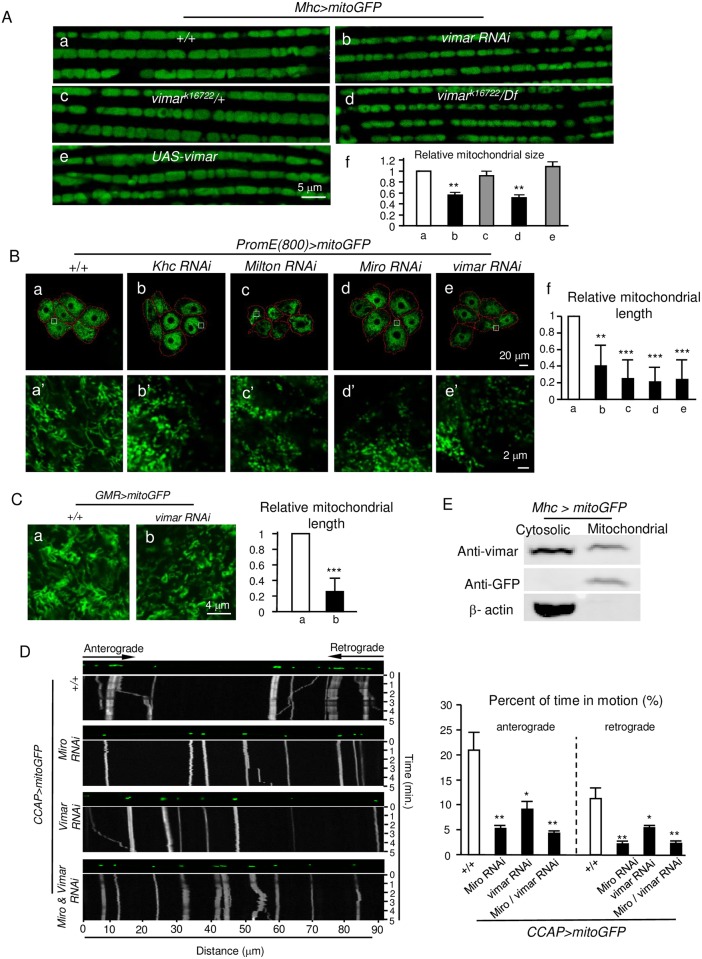
*Drosophila vimar* affects mitochondrial morphology under normal conditions. (**A**) **a-e**, Live imaging of the mitochondrial morphology in the flight muscle of adult flies. The mitochondria are labeled with *UAS-mitoGFP* driven by *Mhc-Gal4* (*Mhc>mitoGFP*). The genotype is indicated on each micrograph. **f**, To quantify the mitochondrial size, the averaged mitochondrial size of the control (+/+) is set as 1, and the relative ratios of the other genotypes to the control are shown. Five thoraces from each genotype were quantified. Bar graphs throughout all figures are means ± SD. The white bar represents the control, the gray bar represents no statistical different from the control, and the black bar represents significantly different from the control. * for p<0.05; ** for p<0.01; ***for p<0.001. (**B**) Mitochondrial distribution and morphology in larval oenocytes. **a**, The mitochondria are labeled with *PromE(800)> mitoGFP*. **b-e**, The effects of *Khc*, *Milton*, *Miro* and *vimar RNAi* are shown. The dotted red lines denote the cell boundaries, which were determined by the mitoGFP background. **a'-e'**, Enlarged view of the white box labeled area in the upper panel. **f**, To quantify the mitochondrial length, the averaged mitochondrial length of the control (+/+) is set as 1, and the relative ratios of the other genotypes to the control are shown. Mitochondrial length of five oenocytes was quantified per genotype and shown as means ± SD. (**C**) Live imaging of mitochondria in eye disc after knocking down *vimar* by *GMR>mitoGFP*. Three eye discs were analyzed for each genotype. (**D**) Effect of *vimar* on mitochondrial transport. The mitochondria are labeled with mitoGFP (*CCAP>mitoGFP*), and their movements in the axons were recorded and transformed into kymographs. Mitochondria motion in ten axons from five larvae was analyzed for each genotype. The quantification is shown on the bar graph. (**E**) Subcellular vimar protein distribution by protein fractionation. The proteins from adult thoraces (*Mhc>MitoGFP*) were separated into cytosolic and crude mitochondrial fractions. The vimar protein enrichment was analyzed by immunobloting with the anti-vimar antibody. The mitoGFP protein was detected by the anti-GFP antibody; and β-actin is a cytosolic protein.

To identify novel regulators of mitochondrial morphology, we studied the flight muscle in *Drosophila* adults, because they have a stereotypic distribution of mitochondria in the longitudinal myofibers [34]. To visualize the mitochondria in the muscle cells, a muscle-specific promoter, *Mhc-Gal4*, was used to drive a mitochondria-targeted GFP (*UAS-mitoGFP*); the progenies were referred to as *Mhc>mitoGFP*. The mitochondrial morphology was clearly observed (Fig 1Aa and 1Af). Using these flies, we accidently observed that mitochondrial fission was enhanced when a *vimar* (*vi**sceral* *m**esodermal* *a**rmadillo-**r**epeats*) RNAi was expressed (Fig 1Ab and 1Af). To further confirm the loss-of-function (LOF) effect of *vimar*, we tested *vimar*^*k16722*^, a P-element mutant with the mobile element inserted into the 5’-UTR region of the *vimar* gene. Again, we observed the trend of enhanced mitochondrial fission in the heterozygous *vimar*^*k16722*^ mutant (Fig 1Ac and 1Af). Because the homozygous *vimar*^*k16722*^ mutant was embryonic lethal, we selected a deficient mutant (*Df(2R) ED1612*) covering the *vimar* locus and generated a trans-heterozygous *vimar* (*vimar*^*k16722*^*/Df*) mutant to further test the effect of *vimar*. In these flies, the mitochondria exhibited a stronger fission morphology compared to the heterozygous mutant (Fig 1Ad and 1Af). These results indicate that *vimar* plays a dominant role in regulating mitochondrial morphology in a dosage-dependent manner. To confirm the mitochondrial defect was generated from loss of *vimar*, we tried to rescue *vimar*^*k16722*^*/Df* by *tubulin-Gal4/UAS-vimar* (*tubulin-Gal4* is a ubiquitously expressed promoter). The result showed that the shortened mitochondria in the *vimar*^*k16722*^*/Df* mutant were rescued by *vimar* overexpression (S1A Fig); while overexpression of *vimar* alone did not affect the mitochondrial morphology (Fig 1Ae and 1Af), suggesting that the levels of the vimar protein may be saturated under normal physiological condition. Using a polyclonal antibody of vimar, we confirmed that the protein levels of vimar were reduced in *vimar*^*k16722*^ and *vimar* RNAi, and increased in the *vimar* overexpression line (S1B and S1C Fig).

To examine mitochondrial distribution, we studied *Drosophila* larval oenocytes because of their stereotypical location and morphology. The wild type mitochondria, labeled with *UAS-mitoGFP* driven by an oenocytes-specific promoter, *PromE(800)-Gal4*, were evenly distributed in the cytosol (Fig 1Ba). As positive controls, we knocked down *Khc* (kinesin heavy chain), *Milton* (an adaptor protein to link Khc to mitochondria) and *Miro*, which are the core components of mitochondrial transport machinery [35]. The results showed that mitochondrial spreading was greatly reduced in the cytosol, and resulted in accumulation in the perinuclear region (Fig 1Bb–1Bd). Interestingly, knocking down *vimar* by RNAi showed a similar distribution pattern (Fig 1Be). For mitochondrial morphology, loss of *Khc*, *Milton*, *Miro* and *vimar* resulted in mitochondrial shortening (Fig 1Ba'–1Be' and 1Bf). Similarly, mitochondria in the eye disc of *GMR>mitoGFP/vimar RNAi* was also shortened (Fig 1C). These results suggest that vimar regulates mitochondrial morphology in different cell types, such as muscle, oenocyte and eye disc.

Transport of mitochondria along the axon can be quantified in *Drosophila* neurons *in vivo* [36]. As a positive control, the *CCAP-Gal4>Miro RNAi* line (*CCAP-Gal4* is a promoter labeling a single axon within a neuron bundle) displayed reduced flux of mobile mitochondria in both anterograde (soma to synapse) and retrograde (synapse to soma) transport (Fig 1D). The *CCAP-Gal4>vimar RNAi* line showed a similar result (Fig 1D). RNAi of both *vimar* and *Miro* resulted in a similar reduction of mitochondrial transport as *Miro* RNAi alone (Fig 1D), suggesting vimar and Miro may function in the same pathway.

To test vimar subcellular localization, proteins from the thoraces of adult flies (*Mhc>MitoGFP*) were extracted and separated into cytosolic and mitochondrial fractions. The Western blot data showed that endogenous vimar was present in both cytosol and mitochondria (Fig 1E). Apart from mitochondria, to test whether Vimar can distribute in other subcellular compartments, we fractionized organelles of ER, lysosome and Golgi apparatus, and found that Vimar was also enriched in the ER fraction, as well as in the cytosol (S2A Fig). This result is consistent with reports suggesting that Miro protein is localized and function in the site of mitochondria-ER junction [37, 38].

### Vimar functions through Miro to regulate mitochondrial morphology

We asked whether vimar regulates mitochondrial morphology through controlling the GTP/GDP exchange of Miro, because Miro is a well-known small GTPase that regulates mitochondrial transport and morphology [10, 14].

**Fig 2:**
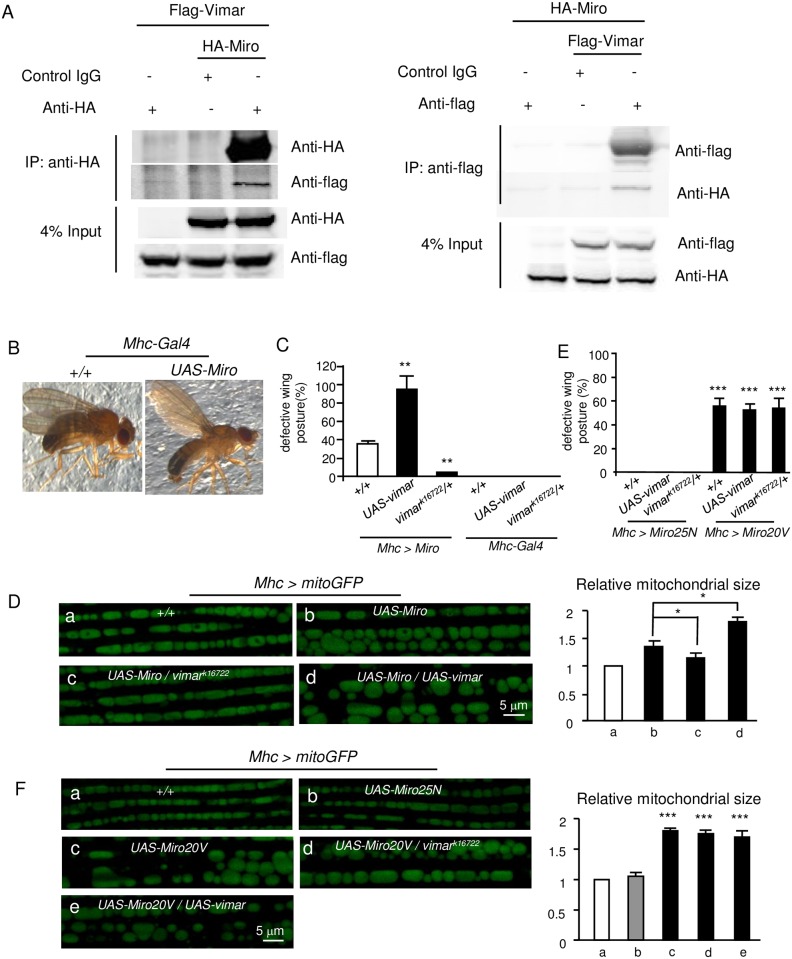
Interaction of vimar and Miro. (**A**) Co-Immunoprecipitation of vimar and Miro. The proteins were collected from the HEK293T cells that expressed both Flag-tagged Vimar (Flag-Vimar) and HA-tagged Miro (HA-Miro). Then, the proteins were precipitated with a HA (left panel) or Flag antibody (right panel). The control IgG is shown as a negative control. The total protein input is shown as the protein loading control. (**B**) An example of the defective wing posture. Compared to the control, overexpression of *Miro* in the adult flight muscle (*Mhc>Miro*) resulted in an upright fly wing posture. (**C**) Quantification of defective wing posture in the *Miro* overexpression background or in the *Mhc-Gal4* background. Trial N = 3, with 100–150 flies examined in each experiment. (**D**) Live imaging of the mitochondrial morphology in the fly flight muscle. The genotype of each fly muscle is labeled on the micrograph. Five thoraces were quantified for each genotype. (**E**) Quantification of defective wing posture in the Miro20V and Miro25N overexpression background. Trial N = 3, with 100–150 flies examined in each experiment. (**F**) Live imaging of the mitochondrial morphology in the fly flight muscle in the indicated genotypes. Five thoraces were quantified for each genotype.

First, we evaluated their physical interactions. The Flag-tagged Vimar (Vimar-Flag) and HA-tagged Miro (Miro-HA) were ectopically expressed in the HEK293T cells. By co-immunoprecipitation (co-IP) assays with anti-HA and anti-Flag antibodies, Miro and Vimar could pull down with each other (Fig 2A). This result suggests that Miro and Vimar can bind with each other, at least under the overexpression conditions.

Next, we tested their genetic interactions. The wing posture defects underline dysfunctional flight muscles that control wing position and movement [39]. It has been reported that overexpression of Miro induces mitochondrial enlargement [13, 15, 16]. Consistently, we observed this mitochondrial change in the Miro overexpression condition. Meanwhile, the wing posture defects of *Mho>Miro* flies increased progressively after eclosion and reached the maximum to approximately 30% at the seventh day after eclosion (Fig 2B and 2C). Interestingly, *vimar*^*k16722*^ almost completely abolished the wing defect induced by the *Miro* overexpression; while *vimar* overexpression greatly enhanced the wing posture defect. As controls, vimar overexpression alone or vimar mutant (*vimar*^*k16722*^) had no wing posture defect (Fig 2C). For the mitochondrial morphology in the *Mhc>mitoGFP* flies, *Miro* overexpression resulted in aberrant mitochondrial size enlargements (Fig 2Da and 2Db), and these defects could be rescued by the heterozygous *vimar*^*k16722*^ mutant (Fig 2Dc). Moreover, *vimar* overexpression further enhanced mitochondrial size increase under the *Miro* overexpression background (Fig 2Dd). These results suggest that *vimar* may genetically interact with *Miro*.

We cannot test effect of GOF *vimar* under the *Miro RNAi* background, because *Miro RNAi* did not induce the wing posture defects in the flight muscles. To further test Miro/vimar interaction, we generated transgenes of constitutively GDP-bound or GTP-bound mutant of Miro. The rational is that GOF or LOF *vimar* should not affect these mutant phenotypes if vimar functions as a Miro GEF. Based on a previous report [40], the amino acid substitutions of A20V (Miro20V) and T25N (Miro25N) should render Miro constitutively GTP-bound and GDP-bound, respectively. As expected, a Miro25N overexpression in the flight muscle (*Mhc>Miro25N*) did not affect the wing posture (Fig 2E) or mitochondria morphology (Fig 2Fa and 2Fb). In contrast, a strong wing posture defect (Fig 2E) and enlarged mitochondria size (Fig 2Fc) were observed in the Miro20V overexpression line (*Mhc>Miro20V*). Importantly, GOF or LOF *vimar* failed to affect the defects in the Miro20V overexpression line (Fig 2E, 2Fd and 2Fe). We also examined vimar effect on mitochondrial transport in the GOF *MiroWT*, *Miro20V* and *Miro25N* background. However, we found that almost no mitochondria were distributed in the axons in the GOF MiroWT or Miro20V background. This data is consistent with previous reports indicating GOF Miro strongly increased mitochondrial length and reduced transportation [41, 42]. We could not examine their mitochondrial transports. In contrast, mitochondrial transport was unaltered under GOF vimar background or combined with Miro25N expression (S2B Fig). Together, these results suggest that vimar requires the normal GTP/GDP binding activity of Miro for its function.

To test whether the vimar/Miro interaction depends on the GTPase activity of Miro, we co-transfected vimar-Flag with inactive (Miro25N) and active (Miro20V) form of *Drosophila* HA-Miro in the HEK293T cells. The co-IP results showed that the vimar/Miro interaction was unaffected by these Miro mutants (S2C Fig). This result suggests that Miro/vimar interaction is not regulated by the GTPase activity of Miro. For mitochondrial distribution of vimar under the LOF *Miro* background (*Mhc>mitoGFP/Miro RNAi*), we observed that the mitochondrial fraction of vimar was unaltered (S3A Fig). This result indicates that vimar may attach with mitochondria by itself or with other partners.

It has been reported that mitochondrial shortening caused by Miro loss required the function of Drp1 [16]. Therefore, we could expect that loss of Drp1 might rescue the mitochondrial shortening in the muscle of *Miro* RNAi background. Indeed, it is the case (S3B Fig). Regarding the interaction between Drp1 and Vimar, our data showed that loss of Drp1 also rescued the mitochondrial shortening of the Vimar mutant (S3B Fig). This result indicates that Miro/Vimar complex is likely to regulate mitochondrial fission through Drp1.

To study whether Miro/vimar affected the Drp1 recruitment to mitochondria under *Miro RNAi* or *vimar RNAi* backgrounds, we used a transgene with a 9.35 kb genomic DNA insertion, which contains an endogenous Drp1 gene labeled by a HA tag (Flag-FlAsH-HA-Drp1) [43, 44]. The result showed that the mitochondria fraction of Drp1 monomer was unaltered in these RNAi conditions (S3C and S3D Fig). This result indicates that loss of Miro/vimar may not affect the recruitment of Drp1 to mitochondria, and how Miro/vimar affects Drp1 function is unclear.

### Vimar promotes mitochondrial fission in response to high calcium concentrations

Miro plays distinct roles in regulating mitochondrial morphology under normal and high calcium conditions [16]. In normal conditions, Miro increases mitochondrial size through inhibition of Drp1 function [13, 15, 16]; however, it promotes mitochondrial fission in high calcium conditions by increasing Drp1 activity, such as in depolarized neurons [16, 35]. If vimar functions through Miro, we expect that vimar may promote mitochondrial fission in high calcium conditions.

To test Miro/vimar response at high calcium state, We had previously established a fly model to study the high calcium-induced cellular response, and accomplished calcium overload by expressing a leaky cation channel, the glutamate receptor 1 Lurcher mutant (GluR1^Lc^) [45, 46]. This fly model (simplified as the *AG* model) contained *A**ppl-Gal4* (a neuron-specific promoter), *UAS-**G**luR1*^*Lc*^ and *tub-Gal80*^*ts*^ (an inhibitor of Gal4 at 18°C, which lost its function at 30°C). Thus, the *AG* flies were normal at 18°C, and calcium overload was induced upon a shift to 30°C [45]. Following the time progression after the GluR1^Lc^ induction, calcium accumulates and neuronal necrosis increases gradually in the *AG* flies [45].

**Fig 3:**
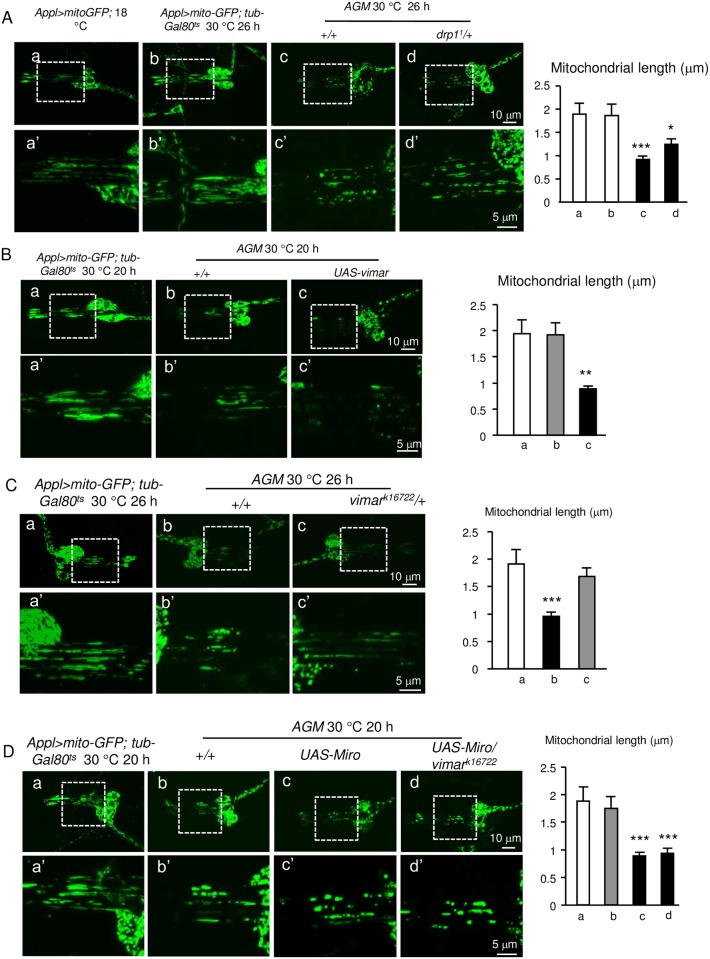
Vimar modulates the mitochondrial length in the high calcium condition. (**A**) Effect of the Drp1 mutation on mitochondrial fission in high calcium conditions. **a** and **b**, Mitochondrial dendrites in the larval chordotonal neurons in the control flies (*Appl>mitoGFP* at 18°C and *Appl>mitoGFP*, *tub-Gal80*^*ts*^ at 30°C). **c** and **d**, Mitochondrial morphology in the *AGM* and *AGM/drp1*^*1*^ flies. **a'-d'** are the enlarged view from the boxed area in **a-d**, and the mitochondrial lengths in **a'-d'** were quantified. 10 to 16 chordotonal organs from each genotype were examined. (**B**) Effect of the *vimar* overexpression on the mitochondrial length after induction of *AGM* expression for 26 hours. 10 to 16 chordotonal organs were examined for each genotype. (**C**) Effect of *vimar* mutant (*vimar*^*k16722*^) on the mitochondrial fragmentation of the *AGM* flies. 10 to 16 chordotonal organs were examined for each genotype. (**D**) Effect of *Miro* overexpression and the *vimar* mutant on the mitochondrial fragmentation of the *AGM* flies. 10 to 16 chordotonal neurons were examined for each genotype.

It is well known that mitochondrial fragmentation occurs upon calcium overloaded [47]. To recapitulate this phenomenon and observe mitochondrial morphology by live cell imaging, we added *UAS-mitoGFP* to the *AG* flies (simplified as the *AGM* model). After the *AGM* larval flies were raised at 30°C to induce calcium influx for 20 hours, mitochondrial fragmentation in the chordotonal neurons showed subtle fission compared to control; while at the 26 hour, the mitochondria in the *AGM* dendrites underwent dramatic fragmentation (Fig 3A–3D). For the rescue effect of a given genetic manipulation, we showed the 26 hour time point (to rescue the more severe defects); and for the enhancer effect of a given genetic manipulation, we showed the 20 hour time point (to enhance a less defective phenotype). As a positive control, a LOF mutant of Drp1, *drp1*^*1*^, which possessed an A186V amino acid substitution at the Dynamin-GTPase domain [44], strongly suppressed the mitochondrial fission defect (Fig 3Ac and 3Ad). These results suggest that the *AGM* model can be adopted to study the mitochondrial morphology in high calcium conditions.

Because Miro promotes mitochondrial fission in the high calcium conditions [16], we expected that vimar might enhance Miro function under high calcium concentrations; and the LOF *vimar* might rescue the mitochondrial fission defect in the *AGM* flies. Indeed, GOF *vimar* enhanced mitochondrial fission (Fig 3Bb and 3Bc); and *vimar*^*k16722*^ rescued mitochondrial fission in the *AGM* flies (Fig 3Cb and 3Cc). In the high calcium state, the mitochondrial localization of vimar was unaltered (S4 Fig), indicating recruitment of vimar on mitochondria is likely independent on calcium level. To further test the role of Miro/vimar complex, we examined effect of the GOF *Miro* transgene in the *vimar*^*k16722*^ background. The result demonstrated that the GOF *Miro* enhanced mitochondrial fission (Fig 3Db and 3Dc), whereas *vimar*^*k16722*^ could not rescue the defect (Fig 3Dd), indicating that basal function of Miro may be partially independent from vimar. Together, these results suggest that vimar functions through Miro to regulate mitochondrial morphology in high calcium conditions.

### The loss of *vimar* suppressed both necrotic cell death and muscle defects in a *Drosophila* PD model

**Fig 4:**
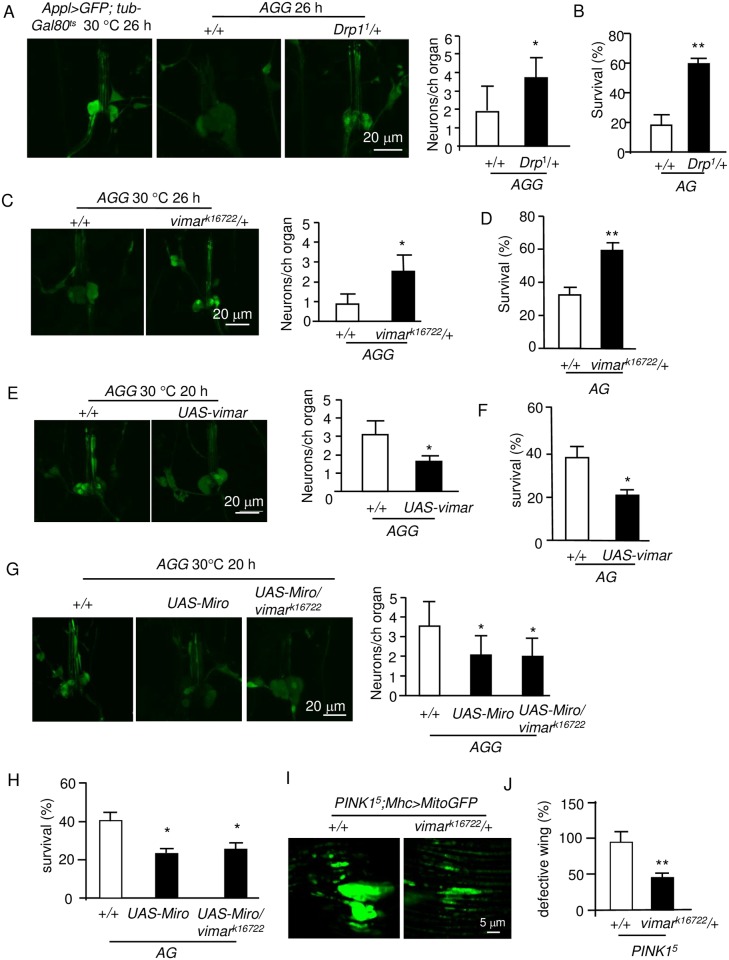
Vimar suppresses neuronal necrosis and muscle degeneration induced by the *Pink1* mutant. (**A**) Effect of the *Drp1* mutant on neuronal necrosis. The micrographs showed the live images from larval chordotonal neurons. The control (*Appl>GFP;tub-Gal80*^*ts*^) displays the cell bodies of the wild type chordotonal neurons, which form a cluster containing 6 neurons. In the *AGG* background, the wild type (+/+) flies showed swollen cell bodies, weakened GFP intensity and neuronal cell loss; and these defects were rescued under the *Drp1* mutant (*Drp1*^*1*^) background. The right panel shows the quantification of the cell loss. For all quantification of neuronal necrosis, trial N = 5, with 10–15 flies were examined in each trial in this figure. (**B**) Effect of the *Drp1* mutant on the survival of the *AG* adult flies. For all quantification of *AG* lethality, trial N = 3, with 100–150 flies were examined for each trial. (**C**) Effect of *vimar* mutant on neuronal necrosis. (**D**) Effect of the *vimar* mutant on the survival of the *AG* flies. (**E**) Effect of *vimar* overexpression on neuronal necrosis. (**F**) Effect of *vimar* overexpression on the survival of the *AG* flies. (**G**) Effect of *Miro* overexpression on neuronal necrosis. The result showed that *Miro* overexpression enhanced neuronal necrosis; and the *vimar* mutant had no rescue effect on this defect. (**H**) Effect of *Miro* overexpression on the survival of the *AG* flies. (**I**) Effect of the *vimar* mutant (*vimar*^*k16722*^) on *PINK1* mutant induced mitochondrial defect. The live image showed the mitochondrial morphology in the *PINK1* mutant (*PINK1*^*5*^) and under the *vimar* mutant background. Ten thoraces were analyzed for each genotype. (**J**) Effect of the *vimar* mutant (*vimar*^*k16722*^) on the wing posture defect of the *PINK1* mutant (*PINK1*^*5*^). Trial N = 3, with 100–150 flies were examined in each trial.

Mitochondrial fission may enhance calcium overload-induced necrotic cell death in neuron cultures [47]. However, there is still insufficient genetic evidence to demonstrate that mitochondrial fission plays a causal role in neuronal necrosis [48]. To study this question, we previously showed that we could quantify necrosis in the *AGG* flies (the *AG* flies containing *UAS-GFP*) at single cell resolution [45]. The result showed that *Drp1*^*1*^ could rescue necrosis in the chordotonal neurons (Fig 4A). In addition, the function of these neurons could be assessed at the behavioral level by quantifying adult fly death [45]; *Drp1*^*1*^ rescued the lethality of the *AG* flies (Fig 4B). Strikingly, *vimar*^*k16722*^ exhibited a rescue effect in the *AGG* flies at both the cellular and behavioral levels (Fig 4C and 4D). In contrast, the GOF *vimar* transgene had the opposite effect (Fig 4E and 4F). This result is consistent with the suppression of mitochondrial fission in this mutant. Furthermore, the GOF *Miro* transgene enhanced necrosis; however, *vimar*^*k16722*^ did not rescue the GOF *Miro* phenotype (Fig 4G and 4H), similar to its effect on mitochondria. These results indicate that Miro has the dominant role in the Miro/vimar complex and that the Miro/vimar complex plays a functional role in neuronal necrosis.

In the *PINK1* mutant of the Parkinson's disease (PD) *Drosophila* model, mitochondrial fusion is enhanced, and the LOF *Miro* mutant could suppress this mitochondrial defect [13]. Therefore, we speculate that the LOF *vimar* mutant might rescue the defective mitochondrial fusion in the *PINK1* mutant. To test this hypothesis, we studied a *PINK1* mutant, *PINK1*^*5*^ [49]. In the *PINK1*^*5*^ flies, the mitochondria are abnormally elongated and fused (Fig 4I), and the fly wing posture is defective (Fig 4J). Strikingly, *vimar*^*k16722*^ could rescue both the mitochondrial morphology and wing posture defects (Fig 4I and 4J). Together, these results indicate that LOF of the Miro/vimar complex suppressed both mitochondrial fragmentation during necrosis and *PINK1* mutant of *Drosophila* PD model.

Furthermore, we found that *vimar*^*k16722*^ and *UAS-vimar* had no effect on classical apoptosis induced by Hid expression [50] (S5 Fig), suggesting that vimar may specifically affect PD and necrosis, but does not regulate apoptosis.

### The mammalian homolog of vimar (RAP1GDS1) plays a similar role in mitochondrial morphology and cell death

**Fig 5:**
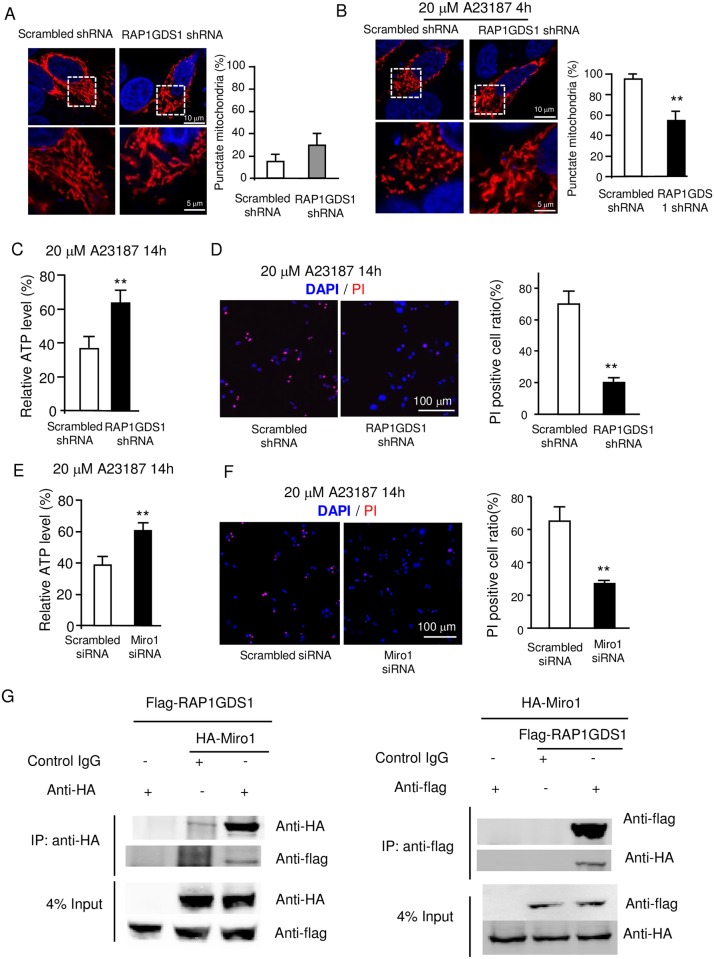
The conserved role of RAP1GDS1 in mammalian cells. (**A**) Effect of RAP1GDS1 knock down on the mitochondrial morphology in HEK293T cells. The mitochondria in the cells that stably express the RAP1GDS1 shRNA are labeled with a transiently transfected MitoDsred expression vector. The cells were classified as tubular-shape or punctate-shape based on differences in their mitochondrial lengths. The ratio of punctate-shape mitochondria is shown in the right panel. The result showed that RAP1GDS1 shRNA had a trend to increase the punctate-shape mitochondria (not statistically different from the control shRNA). Trial N = 3, with 100 cells were quantified in each trial. (**B**) Effect of RAP1GDS1 knocking down on the mitochondrial fragmentation under calcium overload stress. The HEK293T cells were treated with 20 μM calcium ionophore (A23187) for 4 hours. The result showed that RAP1GDS1 shRNA reduced fragmented mitochondria upon calcium ionophore treatment. Trial N = 3, with 100 cells were quantified in each trial. (**C**) Effect of the RAP1GDS1 shRNA on calcium ionophore-induced necrosis. The HEK293T control and RAP1GDS1 shRNA stable cell lines were treated with 20 μM A23187 for 14 hours. Then, the cell death was quantified by the ATP assay. The result indicated that less cell death occurred in the RAP1GDS1 shRNA expressing cells. Trial N = 3. (**D**) Effect of the RAP1GDS1 shRNA on calcium ionophore-induced necrosis. The PI and DAPI staining patterns are shown. The red signals indicate the PI-positive cells and the blue channel indicates the DAPI staining. Trial N = 3. (**E**) Effect of the *Miro1* siRNA on calcium ionophore induced necrosis determined by the ATP assay. The *Miro1* siRNA was transiently transfected in HEK293T cells for 48 hours. Trial N = 3. (**F**) Effect of the *Miro1* siRNA on calcium ionophore induced necrosis determined by the PI staining assay. The PI and DAPI staining patterns are shown. The same result was observed as in **E**. Trial N = 3. (**G**) Co-Immunoprecipitation of RAP1GDS1 and Miro1. The proteins were collected from the HEK293T cells that expressed Flag-tagged RAP1GDS1 (Flag-RAP1GDS1) and HA-tagged Miro1 (HA-Miro1). The control IgG is shown as a negative control. The total protein input is shown as the protein loading control. Trial N = 3.

A protein sequence comparison showed that *Drosophila* vimar shares great similarity with the mammalian protein RAP1GDS1 (S6 Fig); however, it is not clear whether vimar is a functional homolog of RAP1GDS1 [51]. Here, we further investigated the role of RAP1GDS1 in mitochondrial morphology. First, we used a lentivirus to transfect a RAP1GDS1 shRNA into HEK293T cells and established a stable cell line. As expected, the protein level of the RAP1GDS1 was significantly reduced in the shRNA line (S7A Fig). Then, this shRNA line was transiently transfected with a mitochondrial reporter, mitoDsRed. We found that the mitochondrial length showed a trend of reduction in the RAP1GDS1 shRNA cells (Fig 5A). Next, we studied the effect of RAP1GDS1 on necrosis. Necrotic cell death was induced by a calcium ionophore (A23187), which causes calcium overloading and necrosis [52]. As expected, the calcium ionophore induced mitochondrial fragmentation, and the RAP1GDS1 shRNA rescued the mitochondrial defect (Fig 5B). To quantify the cell death, we measured cellular ATP level and performed propidium iodide (PI) staining [53]. The result showed that RAP1GDS1 shRNA rescued necrosis in both assays (Fig 5C and 5D). Moreover, we tested the RAP1GDS1 shRNA in another human cell line, the SH-SY5Y neuroblastoma cells. Similar to the HEK293T cells, the RAP1GDS1 shRNA protected the SH-SY5Y cells from calcium overload (S7B and S7C Fig). In addition, we examined the effect of a Miro-1 siRNA on calcium ionophore induced necrosis. The result showed that it also rescued the cell death (Fig 5E and 5F, and the Miro-1 siRNA effect is shown in S7D Fig). Furthermore, the HA-tagged Miro1 and the Flag-tagged RAP1GDS1 could co-immunoprecipitate *in vitro* (Fig 5G). Together, these results indicate that the function of the Miro1/RAP1GDS1 complex in regulating mitochondrial morphology and necrosis is conserved with the *Drosophila* Miro/vimar complex.

## Discussion

### Vimar is a novel regulator of mitochondrial morphology

Mitochondrial function can be assessed by the enzymatic activity of citrate synthase (CS), the first enzyme in the Krebs cycle that converts acetyl-CoA and oxaloacetate to citrate [54]. In cultured *Drosophila* S2 cells, *vimar* knock down by RNAi resulted in reduced CS activity [54], indicating that vimar may positively regulate mitochondrial function. Because mitochondrial fission has generally been associated with reduced mitochondrial respiration [55], the decreased CS activity may be a result of mitochondrial fission. Consistent with this notion, our results demonstrated that the LOF of *vimar* promoted mitochondrial fission. In addition, a GOF *vimar* transgene had a minimal effect on mitochondrial morphology, indicating that vimar activity might be saturated under normal physiological conditions.

### Vimar functions through Miro to regulate mitochondrial morphology

Because Vimar has been predicted to be a GEF, we hypothesized that vimar may regulate mitochondrial morphology by affecting a small GTPase, which requires a GEF to help with the GTP/GDP exchange process [19]. Interestingly, Miro is one such small GTPase that is known to play important roles in mitochondrial fission and transport [10, 14, 16]. We propose that vimar and Miro may function as a complex. First, a fraction of the vimar protein was localized to the mitochondria, possibly indicating a functional role on mitochondria. Interestingly, the mitochondrial localization of vimar seems not dependent on Miro, because LOF Miro did not affect the mitochondrial fraction of vimar. This indicates that vimar may directly bind with mitochondria or through other scaffolding proteins. Second, vimar and Miro could physically interact with each other, at least *in vitro*. Their interaction seems not affected by the GTPase activity of Miro, because the constitutively GDP- or GTP-bound Miro mutants did not affect their interactions. Third, vimar genetically interacted with Miro. This included the result demonstrating that the LOF *vimar* mutant reduced the effect of Miro on mitochondrial fission inhibition and the GOF vimar transgene had the opposite effect. Moreover, in the constitutive GFP-bound or GDP-bound Miro mutants, the effect of the GOF or LOF *vimar* was abolished. Therefore, vimar requires the normal GDP/GTP binding activity of Miro to function. It is also known that Miro1 overexpression increase mitochondrial size partially by suppression of the Drp1 function [15, 16]. Consistently, increased mitochondrial fission in the LOF of Miro or vimar was abolished by loss of Drp1, suggesting the Miro/vimar complex depends on Drp1 to regulate mitochondrial morphology.

### The Miro/vimar complex may regulate PD and neuronal necrosis through mitochondrial fusion and fission

Familial PD caused by mutations in *PINK1* or *Parkin* results in a series of mitochondrial dysfunctions, particularly the failure to eliminate damaged mitochondria through mitophagy [56, 57]. In these *PINK1* or *Parkin* mutants, the key proteins involved in mitochondrial fusion and fission, such as Marf/Mitofusin and Miro, accumulate [13, 58]. In the *PINK1* mutant flies, the flight muscle is damaged, resulting in wing posture defects [59]. Similarly, we observed that Miro overexpression in the flight muscle resulted in a strong wing posture defect. This result may explain the wing posture defect in the *PINK1* mutant, in which the levels of the Miro protein are increased [13]. Our result demonstrated that the LOF of *vimar* could rescue the wing defect in the *PINK1* mutant, consistent with the hypothesis that vimar functions through Miro.

**Fig 6:**
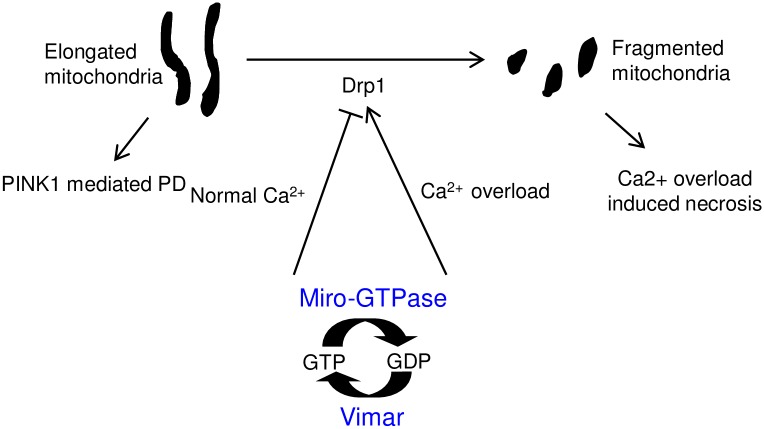
A schematic model of Miro/vimar function on mitochondrial morphology. In normal calcium conditions, the Miro/vimar complex promotes mitochondrial fission inhibition, and their GOF results in elongated mitochondria. Increased mitochondrial fusion is known to occur in the *PINK1* mutant flies, and this defect can be rescued by LOF Miro/vimar. In the high calcium state, the Miro/vimar complex promotes mitochondrial fragmentation, which accelerates neuronal necrosis. Regardless of the intracellular calcium level, vimar enhances the function of Miro, because vimar is likely the GEF to promote Miro's GTP/GDP exchange.

When the intracellular calcium level is high, Miro switches from promoting mitochondrial fission inhibition to enhancing mitochondrial fission [16]. The mechanism for this switch is unclear, although alterations of Drp1 function could be one possibility [16]. Interestingly, Gem1, the yeast homolog of Miro GTPase, has been reported to function as a negative regulator for ER-mitochondria contacts, where Drp1 aggregates and cleaves mitochondria into smaller units [37]. This may serve as the mechanism for Miro to regulate mitochondrial morphology via Drp1. In addition to affect mitochondrial fission, Miro also regulates mitochondrial transport in a calcium dependent manner. For mitochondrial transport, Miro forms protein complexes with Milton, a kinesin adaptor, and with motor proteins, such as kinesin and dynein [35]. In high calcium conditions, Miro alters its binding patterns and results in reduced transport activity [27, 60, 61]. Based on these reports, we proposed that the Miro/vimar complex acted together to affect mitochondrial morphology: at normal condition, Miro/vimar inhibits fission via Drp1; at high calcium state, Ca^2+^ bound Miro switches its function to promote fission. Indeed, vimar responds to the calcium change in the same way as Miro (Fig 6). In addition, our data demonstrated that knocking down RAP1GDS1 and Miro1 increased mitochondrial fission and could rescue calcium overload induced necrosis, similar to the loss of vimar or Miro in *Drosophila*. These data support the hypothesis that RAP1GDS1 is the mammalian homolog of vimar, supporting a previous prediction [51].

Mitochondrial fission plays important role in apoptosis by promoting mitochondrial outer-membrane permeabilization (MOMP) to release cytochrome c from the mitochondria [62]. The use of the Drp1 inhibitor mdivi to block fission has been shown to be an effective treatment for stroke [47], and the function of mitochondrial fission on necrotic cell death has been well documented [24, 26, 48]. The uncertainty lies in the lack of genetic evidence and downstream mechanism of mitochondrial fission in necrosis [48]. Our data demonstrated that mitochondrial fragmentation occurred in necrotic neurons, and the LOF *Drp1* and *vimar* mutants both suppressed neuronal necrosis.

Much evidence suggests that the mitochondrial fusion and fission defects are directly linked to many human diseases [22], and strategies that target the Miro/vimar complex may affect a broad spectrum of diseases. For instance, mutations in the fragile X mental retardation 1 (*FMR1*) gene, which result from expansion of trinucleotide repeat in the 5′ untranslated region, often cause enhanced mitochondrial fission and mental retardation syndrome [63]. Likewise, aberrant mitochondrial fusion was observed in a *Drosophila* Alzheimer's disease model induced by the ectopic expression of a human tau mutant (tau^R406W^) [43]. In this case, the tau mutant may promote excessive actin stabilization to decrease Drp1 recruitment to the mitochondria, which results in excessive mitochondrial fusion and neurodegeneration [43, 64]. Due to the dual function of the Miro/vimar complex in high-Ca^2+^ induced necrosis and *PINK1* mutant induced PD, a drug to target this complex may benefit both disease states. As a modulator, it may be safer to target vimar/ RAP1GDS1.

## Supporting Information

S1 Fig (**A**) **a-c**, Live imaging of the mitochondrial morphology in the flight muscle of adult flies. The mitochondria are labeled with *UAS-mitoGFP* driven by *Tubulin-Gal4* (*Tubulin>mitoGFP*). The genotype is indicated on each micrograph. **d**, The averaged mitochondrial size of the control (+/+) is set as 1, and the relative ratios of the other genotypes to the control are shown. Five thoraces from each genotype were quantified. Bar graphs throughout all figures are means ± SD. The white bar represents the control, the gray bar represents no statistical different from the control, and the black bar represents significantly different from the control. * for p<0.05; ** for p<0.01; ***for p<0.001. **(B**) and (**C**) Vimar protein level in the adult thoraces. The Western blot shows immunobloting with a vimar antibody, with the genotype listed on each lane. β-actin is shown as the protein loading control. The quantified data is shown as means ± SD. Trial N = 3.
(PDF)

S2 Fig (**A**) Vimar distribution in other subcellular compartments. The fly homogenate was separated into cytosol, lysosome, Golgi apparatus and ER. Vimar protein level was determined by immunobloting using a vimar antibody. Calnexin, Lamp1, GM130 and β-actin are markers for ER, lysosome, Golgi apparatus and cytoplasm, respectively. (**B**) Effect of *vimar* overexpression on mitochondrial transport. The mitochondria are labeled with mitoGFP (*CCAP>mitoGFP*), and their movements in the axons were recorded and transformed into kymographs. Overexpression of *Miro25N*, *vimar* or both of them had no effects towards mitochondria transport. Mitochondria motion in ten axons from five larvae was analyzed for each genotype. (**C**) Effect of *Drosophila* Miro mutants on its interaction with vimar *in vitro*. The HA-tagged Miro, Miro20V (a constitutive GTP-bound mutant) and Miro25N (a constitutive GDP-bound mutant) was individually co-transfected with Flag-tagged vimar. The co-IP experiment showed that GTP or GDP state of Miro did not affect its interaction with vimar. Trial N = 3. IgG is shown as a negative control. The total protein input is shown as the protein loading control.
(PDF)

S3 Fig (**A**) Effect of LOF Miro on the mitochondrial localization of vimar. In the *Mhc>mitoGFP/Miro RNAi* flies, the mitochondrial fraction of vimar was unaltered as the control *Mho>mitoGFP* flies. Anti-ATP5A is shown as the mitochondria marker and actin as cytosolic marker. Trial N = 2. (**B**) Live image of mitochondria in flight muscle. *Miro RNAi* and *vimar RNAi* resulted in shortened mitochondria, which could be blocked by *Drp1 RNAi*. Five thoraces from each genotype were quantified. (**C**) and (**D**) Drp1 recruitment to mitochondria in *Miro RNAi* or *vimar RNAi* background. Thoracic homogenate was separated into cytosol and mitochondria and Drp1 protein level in different fractions was immunoblotted. Anti-ATP5A is shown as the mitochondria marker and actin as cytosolic marker. Trial N = 2.
(PDF)

S4 Fig Vimar protein distribution in mitochondria fraction under high calcium stress. The *AG* fly heads were homogenized and separated in cytoplasmic fraction and crude mitochondria. Vimar level was labeled by a vimar antibody. Anti-ATP5A is shown as the mitochondria marker and actin as cytosolic marker. Trial N = 2.
(PDF)

S5 Fig Effect of LOF and GOF *vimar* on apoptosis. The apoptotic flies (*GMR-Gal4;GMR-Hid*) showed smaller eye size. Addition of *UAS-P35*, a known apoptosis inhibitor, is shown as a positive control, which rescued the smaller eye size defect. However, *vimar*^*k16722*^ or *UAS-vimar* showed no effect on the eye size defect.
(PDF)

S6 Fig Protein sequence comparison between vimar (635 a.a) and RAP1GDS1 (608 a.a). The alignment is generated from CLUSTAL alignment algorithm.
(PDF)

S7 Fig (**A**) Effect of the RAP1GDS1 shRNA on the level of the RAP1GDS1 protein in the stable HEK293T cells. β-actin was used as the loading control. (**B**) Effect RAP1GDS1 shRNA on necrosis in the stable SH-SY5Y cells. The cells were treated with 20 μM A23187 for 1 hour. The bright field images of the cells showed less cell death in the RAP1GDS1 shRNA cells upon calcium ionophore treatment. Trial N = 4. (**C**) Quantification of necrosis by the ATP assay. The stable SH-SY5Y cell lines were treated with 20 μM A23187 for 6 hours. The result showed that less cell death occurred in the RAP1GDS1 shRNA cells than the control (scrambled shRNA) cells. Trial N = 3. (**D**) The efficiency of the *Miro1* siRNA on *Miro* transcripts in 293T cells. The transcript level of *Miro1* was determined by qRT-PCR. The result showed that *Miro1* siRNA significantly knocked down *Miro1* transcripts. Trial N = 3.
(PDF)
