# Supplementary figures and images for: Vimar Is a Novel Regulator of Mitochondrial Fission through Miro
Source: PLoS Genet. 2016 Oct 7;12(10):e1006359. doi: 10.1371/journal.pgen.1006359 (PMC5065127; doi:10.1371/journal.pgen.1006359)

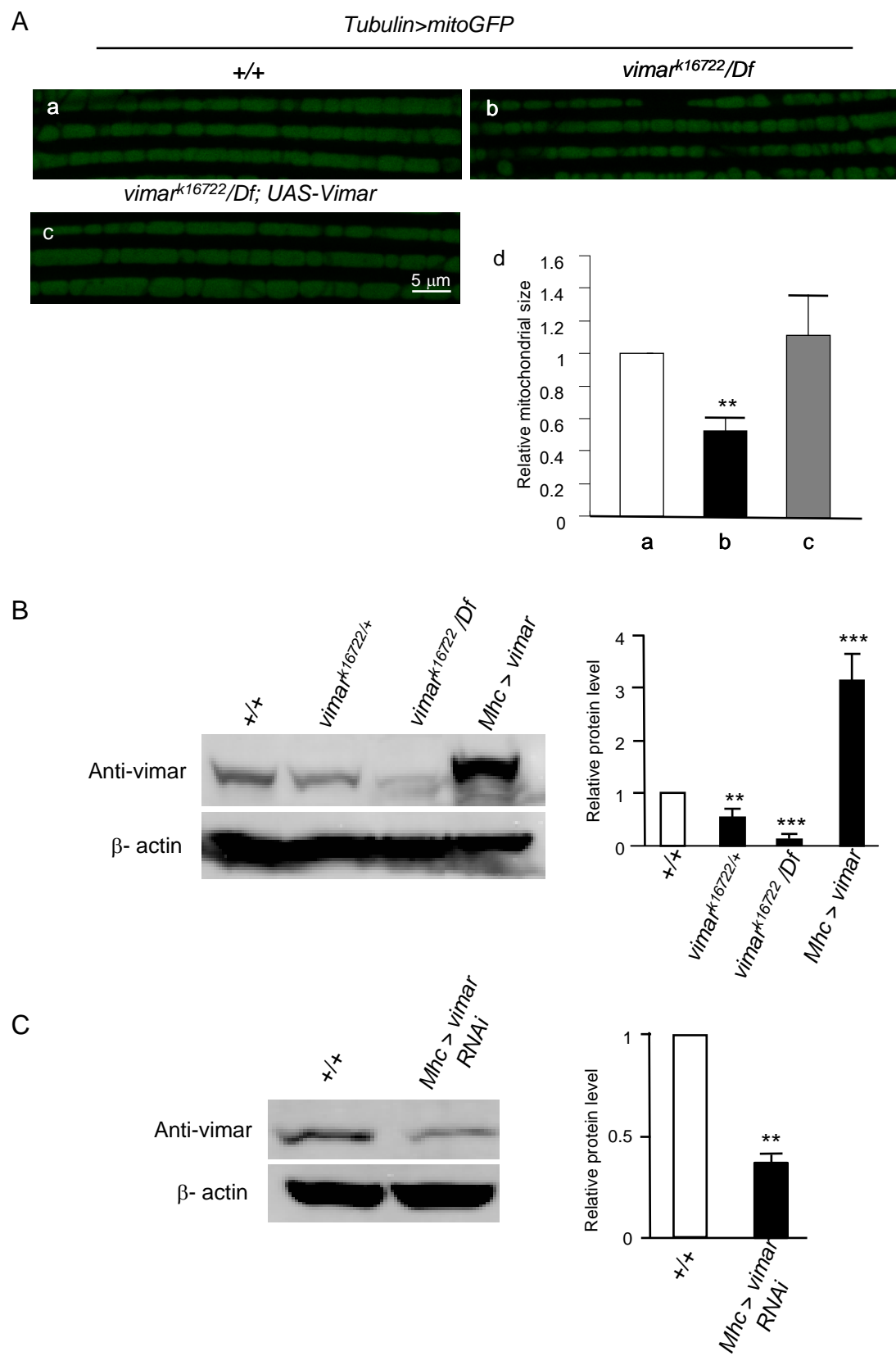

Supplementary Figure 1

Supplement: S1 Fig — (A) a-c, Live imaging of the mitochondrial morphology in the flight muscle of adult flies. The mitochondria are labeled with UAS-mitoGFP driven by Tubulin-Gal4 (Tubulin>mitoGFP). The genotype is indicated on each micrograph. d, The averaged mitochondrial size of the control (+/+) is set as 1, and the relative ratios of the other genotypes to the control are shown. Five thoraces from each genotype were quantified. Bar graphs throughout all figures are means ± SD. The white bar represents the control, the gray bar represents no statistical different from the control, and the black bar represents significantly different from the control. * for p<0.05; ** for p<0.01; ***for p<0.001. (B) and (C) Vimar protein level in the adult thoraces. The Western blot shows immunobloting with a vimar antibody, with the genotype listed on each lane. β-actin is shown as the protein loading control. The quantified data is shown as means ± SD. Trial N = 3. (PDF) [file pgen.1006359.s001.pdf]

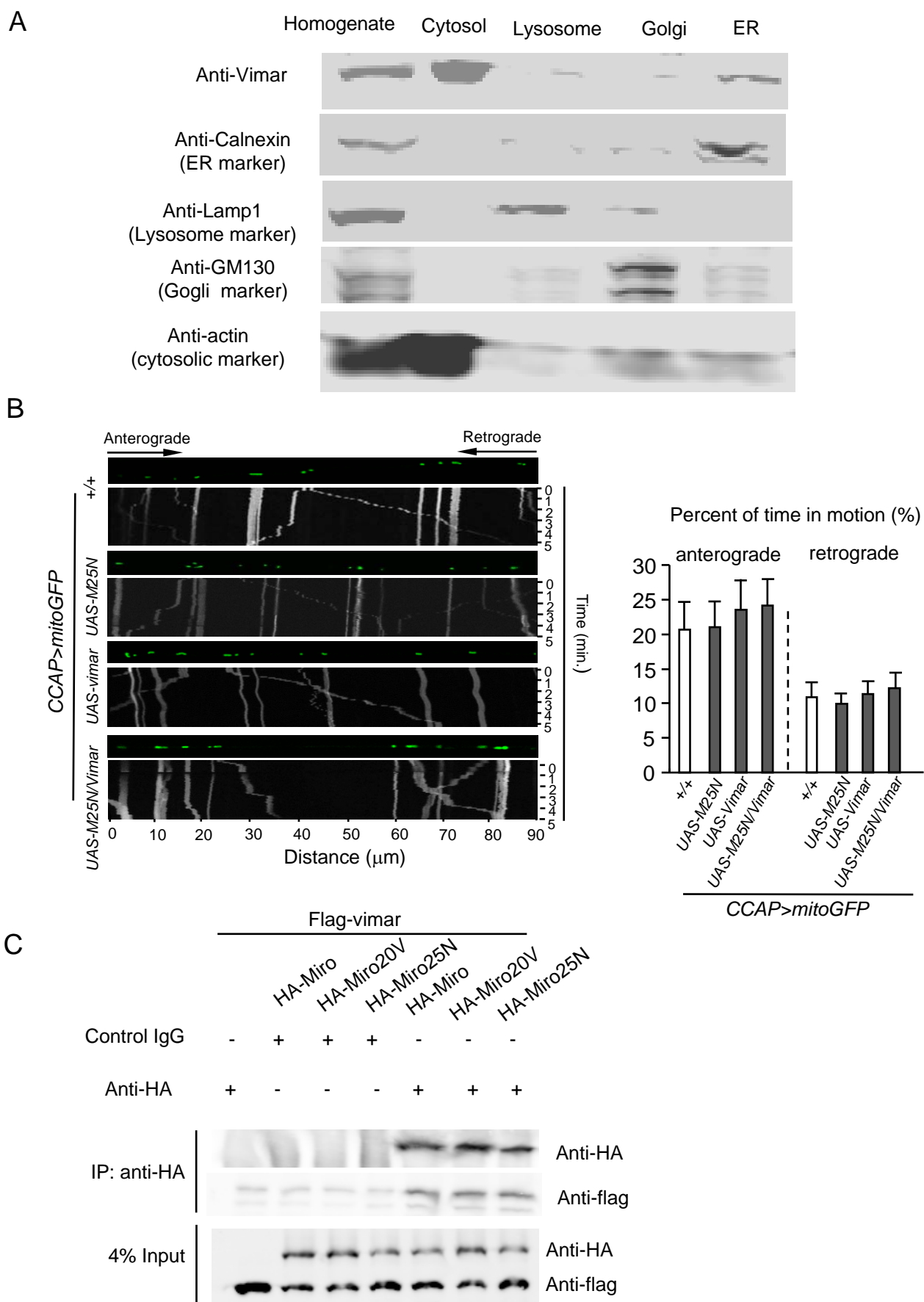

Supplementary Figure 2

Supplement: S2 Fig — (A) Vimar distribution in other subcellular compartments. The fly homogenate was separated into cytosol, lysosome, Golgi apparatus and ER. Vimar protein level was determined by immunobloting using a vimar antibody. Calnexin, Lamp1, GM130 and β-actin are markers for ER, lysosome, Golgi apparatus and cytoplasm, respectively. (B) Effect of vimar overexpression on mitochondrial transport. The mitochondria are labeled with mitoGFP (CCAP>mitoGFP), and their movements in the axons were recorded and transformed into kymographs. Overexpression of Miro25N, vimar or both of them had no effects towards mitochondria transport. Mitochondria motion in ten axons from five larvae was analyzed for each genotype. (C) Effect of Drosophila Miro mutants on its interaction with vimar in vitro. The HA-tagged Miro, Miro20V (a constitutive GTP-bound mutant) and Miro25N (a constitutive GDP-bound mutant) was individually co-transfected with Flag-tagged vimar. The co-IP experiment showed that GTP or GDP state of Miro did not affect its interaction with vimar. Trial N = 3. IgG is shown as a negative control. The total protein input is shown as the protein loading control. (PDF) [file pgen.1006359.s002.pdf]

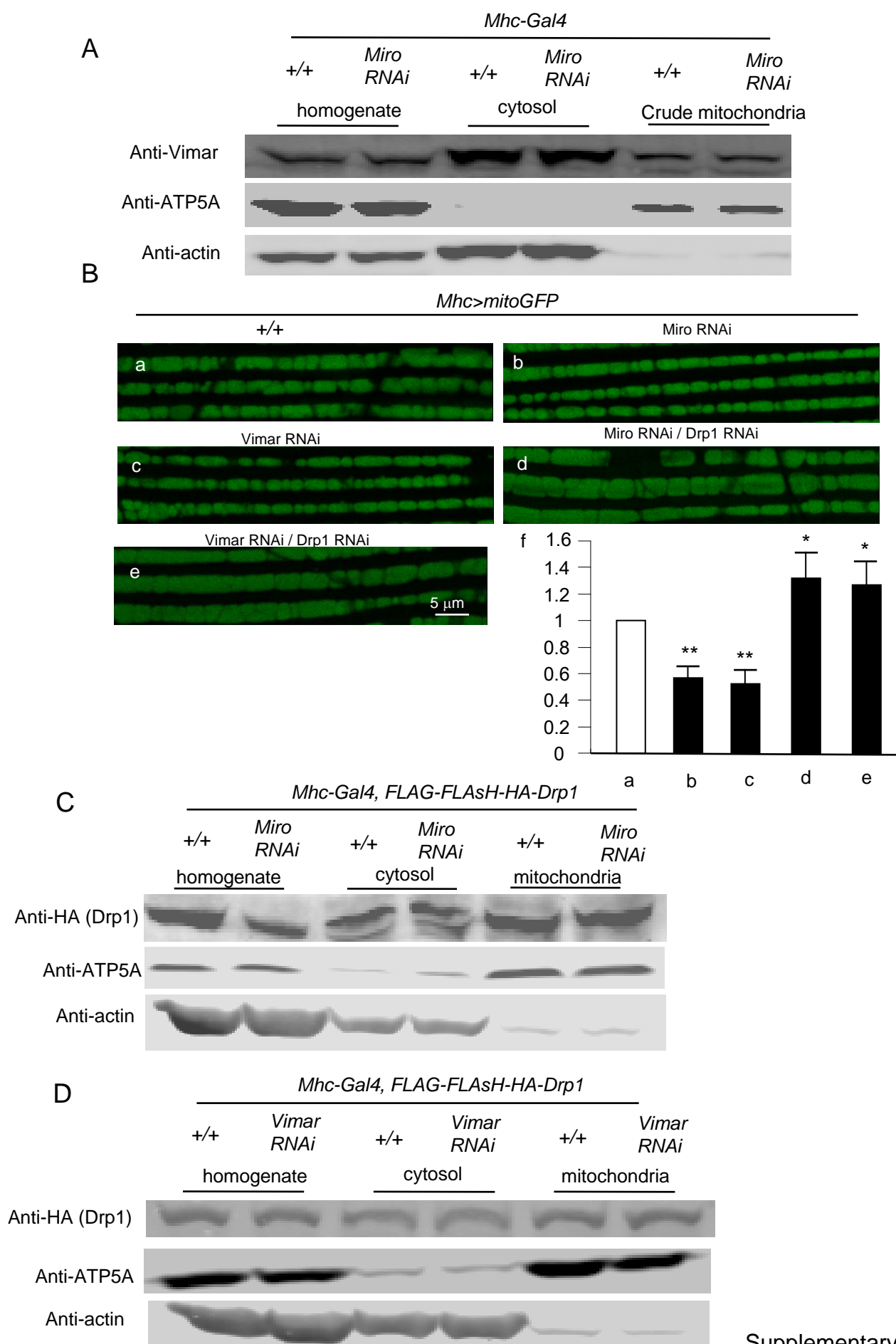

Supplementary Figure 3

Supplement: S3 Fig — (A) Effect of LOF Miro on the mitochondrial localization of vimar. In the Mhc>mitoGFP/Miro RNAi flies, the mitochondrial fraction of vimar was unaltered as the control Mho>mitoGFP flies. Anti-ATP5A is shown as the mitochondria marker and actin as cytosolic marker. Trial N = 2. (B) Live image of mitochondria in flight muscle. Miro RNAi and vimar RNAi resulted in shortened mitochondria, which could be blocked by Drp1 RNAi. Five thoraces from each genotype were quantified. (C) and (D) Drp1 recruitment to mitochondria in Miro RNAi or vimar RNAi background. Thoracic homogenate was separated into cytosol and mitochondria and Drp1 protein level in different fractions was immunoblotted. Anti-ATP5A is shown as the mitochondria marker and actin as cytosolic marker. Trial N = 2. (PDF) [file pgen.1006359.s003.pdf]

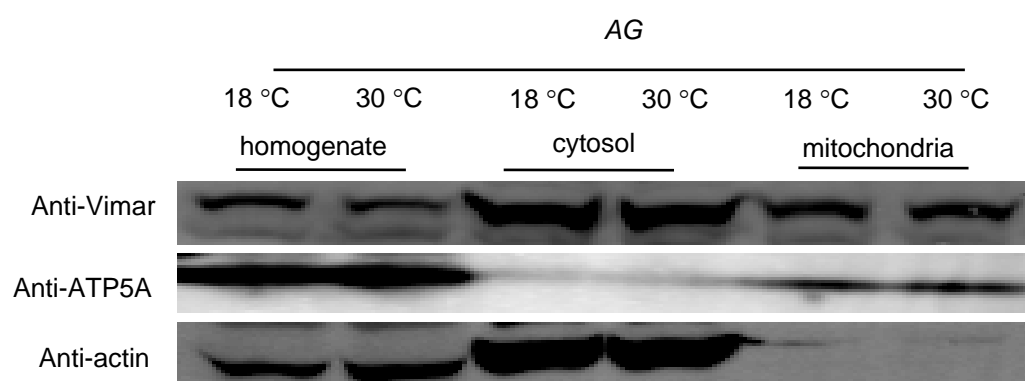

Supplementary Figure 4

Supplement: S4 Fig — The AG fly heads were homogenized and separated in cytoplasmic fraction and crude mitochondria. Vimar level was labeled by a vimar antibody. Anti-ATP5A is shown as the mitochondria marker and actin as cytosolic marker. Trial N = 2. (PDF) [file pgen.1006359.s004.pdf]

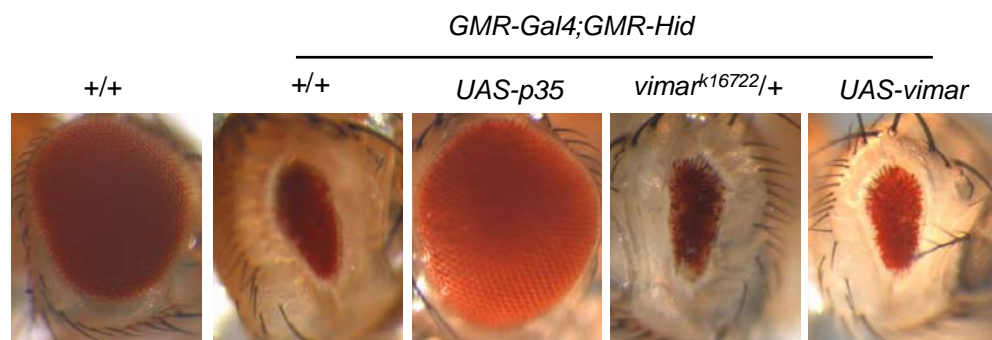

Supplementary Figure 5

Supplement: S5 Fig — The apoptotic flies (GMR-Gal4;GMR-Hid) showed smaller eye size. Addition of UAS-P35, a known apoptosis inhibitor, is shown as a positive control, which rescued the smaller eye size defect. However, vimark16722 or UAS-vimar showed no effect on the eye size defect. (PDF) [file pgen.1006359.s005.pdf]

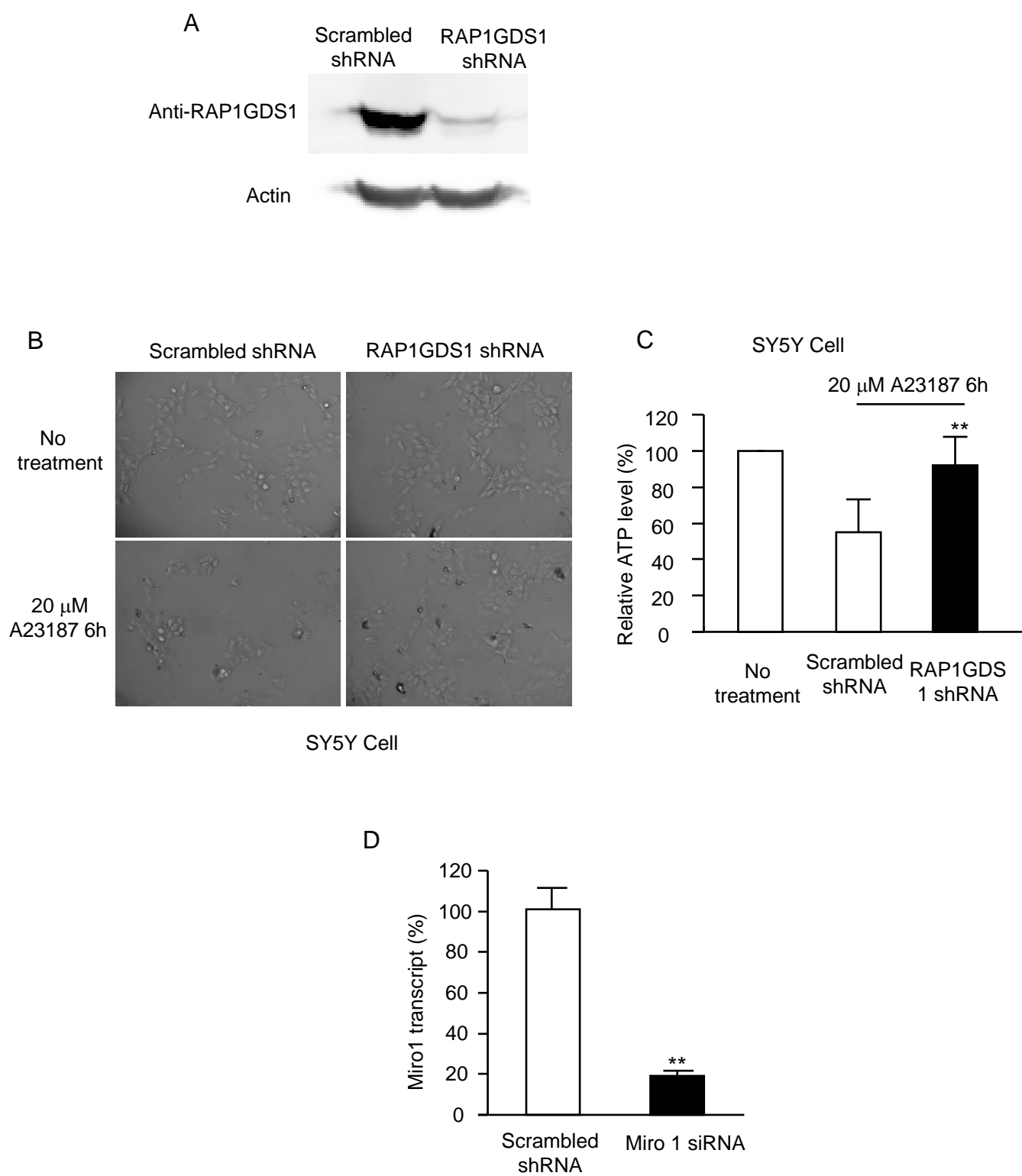

Supplementary Figure 7

Supplement: S7 Fig — (A) Effect of the RAP1GDS1 shRNA on the level of the RAP1GDS1 protein in the stable HEK293T cells. β-actin was used as the loading control. (B) Effect RAP1GDS1 shRNA on necrosis in the stable SH-SY5Y cells. The cells were treated with 20 μM A23187 for 1 hour. The bright field images of the cells showed less cell death in the RAP1GDS1 shRNA cells upon calcium ionophore treatment. Trial N = 4. (C) Quantification of necrosis by the ATP assay. The stable SH-SY5Y cell lines were treated with 20 μM A23187 for 6 hours. The result showed that less cell death occurred in the RAP1GDS1 shRNA cells than the control (scrambled shRNA) cells. Trial N = 3. (D) The efficiency of the Miro1 siRNA on Miro transcripts in 293T cells. The transcript level of Miro1 was determined by qRT-PCR. The result showed that Miro1 siRNA significantly knocked down Miro1 transcripts. Trial N = 3. (PDF) [file pgen.1006359.s007.pdf]
