# Supplementary material for: Vimar Is a Novel Regulator of Mitochondrial Fission through Miro
Source: PLoS Genet. 2016 Oct 7;12(10):e1006359. doi: 10.1371/journal.pgen.1006359 (PMC5065127; doi:10.1371/journal.pgen.1006359)
Supplement: S6 Fig — The alignment is generated from CLUSTAL alignment algorithm. (PDF) [file pgen.1006359.s006.pdf]

```
vimar     —  MATAEIDDLIEKLKTTSVSPANTTNL----------LCEISATKDPKLFDKHELAECFLG
RAP1GDS1  —  -MADNLSDTLKKLKITAVDK-TEDSLEGCLDCLLQALAQNNT-ETSEKIQASGILQLFAS
               : ::.* ::*** *:*.  .  .*          *.: .: : .: ::   : : * .
             LTKCDDTNVRKEAAKCIAEITKSEVQRKKFTKRNIIAFLECLRQVPTSDGSMELPIQIC
             LLT-PQSSCKAKVANIIAEVAKNEFMRIPCVDAGLISPLVQLL-----NSKDQEVLLQTG
             * .  ::. : :.*: ***::*.*. *   .. .:*:.::: *     .. . *: :*
             RALGNICYLNDEARDLILE-LEGDAVLLRLLDITTIEDVANAAQFIKVRGGLLSNYLLGG
             RALGNICYDSHEGRSAVD-QAGGAQIVIDHLRSLCSITDPANEKLLTVFCGMLMNYSNEN
             ******** ..*.*. :     *  :::  *        .   :::.*  *:* **   .
             EGLAKRAMELGVMKKLQGIIDIGASNVEQHEDLLLNTLPLLSILTENVSDLNFDSSLNIQ
             DSLQAQLINMGVIPTLVKLLGIHCQN-AALTEMCLVAFGNLAEL-ESSKEQFASTNIAEE
             :.*  : :::**: .*  ::.* ..*     :: * ::  *: * *. .:   .:.:  :
             LSRILAASTNPDLAEMCLELLHYQAESDEVKLILAKDGLCETIYNLLEKYKTLASTSEAR
             LVKLFKKQIEHDKREMIFEVLAPLAENDAIKLQLVEAGLVECLLEIVQQKVDSDKEDDIT
             * :::  . : *  ** :*:*   **.* :** *.: ** * : :::::     . .:
             ALMKLACELIVLILTGDDSMHYLY---TTPLLKNMVDWLDSTDIDLLTTGVLALGNFART
             EL-KTGSDLMVLLLLGDESMQKLFEGGKGSVFQRVLSWIPSNNHQLQLAGALAIANFARN
              * * ..:*:**:* **:**: *:   . .:::.::.*: *.: :*  :*.**:.****.
             DSHCIYFVEQQTMNKLLEVLAKNNGVKDDVRLQHALLSALRNLVIPKPNKNAVIQAGLVQ
             DANCIHMVDNGIVEKLMDLLDRHVE-DGNVTVQHAALSALRNLAIPVINKAKMLSAGVTE
             *::**::*::  ::**:::* ::   ..:* :*** *******.**  **  ::.**:.:
             TILPMLEIHQPPVVFKLLGTLRMTVDGQEKLALELLKNKTLIEQLVHWSKSSDYAGVTGE
             AVLKFLKSEMPPVQFKLLGTLRMLIDAQAEAAEQLGKNVKLVERLVEWCEAKDHAGVMGE
             ::* :*: . *** ********* :*.* : * :* ** .*:*:**.*.::.*:*** **
             SLRLMAWLIKHAYLSKIAYALPRKGDAPAEQIADKIPLTQDYDRSSLSEFLANEGTVEAM
             SNRLLSALIRHS------------------------------KSKDVIKTIVQSGGIKHL
             * **:: **:*:                              . ..: : :.:.* :: :
             VSMLTAQHLVMQNEALIALCILSVVYLSQPSEAAQ----A----QLLQDELVKCEVGKKL
             VTMATSEHVIMQNEALVALALIAALELGTAEKDLESAKLVQILHRLLADERSAPEIKYNS
             *:* *::*::******:**.:::.: *. ..:  :    .    :** **    *:  :
             AELISK--SSDTMTKEIVE-NLQNCVNLLKSSEQLVAHLEQHNINELLKSIPILTEYCTL
             MVLICALMGSECLHKEVQDLAFLDV------VSKLRSHENKSVAQQASLTEQRLTVES--
               **.   .*: : **: :  : :        .:* :* ::   ::   :   **  .
```

Supplementary Figure 6
